# Senkyunolide A inhibits the progression of osteoarthritis by inhibiting the NLRP3 signalling pathway

**DOI:** 10.1080/13880209.2022.2042327

**Published:** 2022-02-27

**Authors:** Minglei Shao, Dongwei Lv, Kai Zhou, Haijun Sun, Zhitao Wang

**Affiliations:** a Department of Orthopedics, Dongying People’s Hospital, Dongying, PR China; b Department of Joint Surgery, Dongying People’s Hospital, Dongying, PR China; c Department of Orthopedics, Dongying District People’s Hospital, Dongying, PR China

**Keywords:** Chondrocytes, catabolic, anabolic, IL-1β, nigericin

## Abstract

**Context:**

Osteoarthritis (OA) is a degenerative disease. Senkyunolide A (SenA) is an important phthalide from *Ligusticum chuanxiong* Hort (Umbelliferae) with anti-spasmodic and neuroprotective effects.

**Objective:**

We explored the effect of SenA on IL-1β-stimulated chondrocytes and OA mice

**Materials and methods:**

Chondrocytes were stimulated by IL-1β (10 ng/mL) to establish an OA model *in vitro*. Cells were treated with SenA (20, 40, 80 and 160 μg/mL) for 48 h. The *in vivo* OA model was established by cutting off the medial meniscus tibial ligament (MMTL) at right knee incision of male C57BL/6 mice. One week after surgery, mice were injected with SenA (intraperitoneally one week) and divided into four groups (*n* = 6 per group): Sham, OA, OA + SenA 20 mg/kg and OA + SenA 40 mg/kg. The OA progression was examined by haematoxylin and eosin (H&E) staining.

**Results:**

SenA treatment increased cell viability (33%), proliferation (71%), inhibited apoptosis (21%), decreased levels of catabolic marker proteins (MMP13, 23%; ADAMTS4, 31%; ADAMTS5, 19%), increased levels of anabolic marker proteins (IGF-1, 57%; aggrecan, 75%; Col2a1, 48%), reduced levels of inflammation cytokines (TNF-α, 31%; IL-6, 19%; IL-18, 20%) and decreased levels of NLRP3 (21%), ASC (20%) and caspase-1 (29%) of chondrocytes. However, NLRP3 agonist nigericin increased levels of MMP13 (55%), ADAMTS4 (70%), ADAMTS5 (53%), decreased levels of IGF-1 (36%), aggrecan (26%), Col2a1 (25%), inhibited proliferation (61%) and promoted apoptosis (76%).

**Discussion and conclusions:**

SenA alleviates OA progression by inhibiting NLRP3 signalling pathways. These findings provide an experimental basis for the clinical application of drugs in the treatment of OA.

## Introduction

Osteoarthritis (OA) is a degenerative disease involving the structural changes of articular cartilage, ligament, subchondral bone, synovium, capsule and periarticular muscles (Martel-Pelletier et al. 2016). OA is mainly characterized by articular cartilage degeneration, osteophyte formation and subchondral bone thickening, bringing about grievous joint pain and defunctionalization (O'Neill et al. 2018; Charlier et al. 2019). OA strongly impacts a patient's quality of life, has adverse psychological effects, and places a burden on the community (Litwic et al. 2013). At present, most treatments to prevent or slow down the evolvement of OA are ineffective, and OA patients need expensive surgical intervention for arthroplasty (Nganvongpanit et al. 2009; Khan et al. 2018; Abramoff and Caldera 2020). Furthermore, ageing, deficiencies of vitamin and mineral, and drug abuse result in a conspicuously increased risk of OA progression (Rahmati et al. 2017; Wei et al. 2019). Therefore, it is very important to find new molecules used for OA management and elucidate their molecular mechanisms, which will bring new prospects for OA treatment.

Chondrocytes are the unique stationary cells in the bone and joint system, and undergo a large number of changes during OA progression, such as proliferation and secretion (Charlier et al. 2019). The key role of chondrocytes is to maintain anabolic and catabolic homeostasis in the extracellular matrix (ECM) (Si et al. 2017). The aberrant apoptosis, inflammatory response of chondrocytes is related to the matrix degradation and cartilage destruction in OA (Wang et al. 2017). Interleukin-1β (IL-1β), a pro-inflammatory cytokine, can trigger inflammation and the production and secretion of catabolic factors, and play an important part in the pathogenesis of OA (Wang F et al. 2019). Inflammation and loss of ECM are increasingly considered to be important driving factors of OA cartilage damage (Chan et al. 2015; Wang et al. 2020). Therefore, exploring the mechanism of chondrocyte dysfunction will help to provide an understanding of the pathogenesis of OA.

Herbal medicine has been used to treat OA worldwide (Cameron and Chrubasik 2014). These traditional herbal medicines have attracted much attention in the treatment of OA due to their low toxicity and anti-inflammatory biological effects. Hyperoside (Sun et al. 2021), *Paeonia lactiflora* Pall (Ranunculaceae) (Zhu et al. 2019), *Scutellaria baicalensis* Georgi (Lamiaceae) (Khan NM et al. 2017), *Zingiber officinale* Rosc (Zingiberaceae) (Ruangsuriya et al. 2017), *Nigella sativa* L. (Ranunculaceae) (Salimzadeh et al. 2017), *Phyllantus* (Euphorbiaceae) species (Buddhachat et al. 2017), *Phyllanthus amarus* Schum (Euphorbiaceae) (Pradit et al. 2015), chuanxiong (Ye et al. 2011) and others (Ruamrungsri et al. 2016) have been reported to play an anti-inflammatory role in OA. Senkyunolide A (SenA) is an important phthalide in *Ligusticum chuanxiong* Hort (Umbelliferae) (Chan et al. 2007). In recent years, SenA received increasing attention for its anti-spasmodic and neuroprotective effects (Gong et al. 2018; Zheng et al. 2018). However, the therapeutic effect of SenA on OA has never been reported. Studies reported that the NLRP3 inflammasome generates degradative enzymes and pro-inflammatory cytokines (such as IL-1β and TNF-α) to participate in the pathogenesis of many arthritic diseases (McAllister et al. 2018; An et al. 2020). The study of Jia et al. (2021) described the relationship between NLRP3 and risk factors of OA, OA pain, synovitis and cartilage degeneration in detail. Furthermore, it has been reported that Chinese herbal medicine could alleviate OA through restraining NLRP3-mediated inflammatory necrosis (Zu et al. 2019).

However, few studies reported the molecular mechanisms of SenA inhibiting OA and its relationship to NLRP3 signalling pathway. In this study, we found that SenA treatment could inhibit OA *in vivo* and *in vitro* and elucidated the related molecular mechanism as the NLRP3 inflammasome-related signalling pathway.

## Materials and methods

### Animals

Male C57BL/6 mice (200–220 g) were purchased from the Animal Center of the Chinese Academy of Sciences. The mice had free access to water and food. All experimentation on animals were ethically ratified by the Animal Use and Care Committee of Dongying People's Hospital (DYRM20201205), and performed in conformity to the National Institute for Health Guide for the Care and Use of Laboratory Animals.

### Isolation and culture of mice chondrocytes

Primary chondrocytes of mice were isolated and cultured according to a previous protocol (Gosset et al. 2008). Chondrocytes were obtained from femoral head articular cartilage of healthy 5- or 6-day-old male C57Bl/6 mice. The cartilage tissues of mice knee joint were cut into small pieces (<1 mm^3^), digested with 0.25% trypsin (10 mL) for 45 min at 37 °C under 5% CO_2_ condition in a culture dish, and then placed in 0.25% collagenase D solution (10 mL, Thermo Fisher Scientific, Carlsbad, CA) overnight at 37 °C. After centrifugation, chondrocytes were collected and cultured in Dulbecco’s modified Eagle’s medium/Ham Nutrient Mixture F12 (DMEM/F12) medium containing 10% foetal bovine serum (FBS, Thermo Fisher Scientific, Carlsbad, CA), 100 μg/mL streptomycin and 100 U/mL penicillin G (Thermo Fisher Scientific, Carlsbad, CA) at 37 °C under 5% CO_2_ condition, and the medium was changed every 2–3 days. The chondrocytes were passaged 2–3 times when reaching 80% confluence and moved to a culture flask for further experiment.

### Cell treatments

Chondrocytes were stimulated with IL-1β (Xu and Xu 2017) (10 ng/mL, Sigma-Aldrich, St. Louis, MO) for 24 h to simulate an inflammatory response. For the SenA treatment groups, chondrocytes were pre-treated with different concentrations of SenA (0, 20, 40, 80 and 160 μg/mL, MedChemExpress, Monmouth Junction, NJ) for 2 h in serum free medium. To investigate the effects of SenA on IL-1β-induced chondrocytes, the chondrocytes were divided into five groups: control group (untreated cells), IL-1β, IL-1β + SenA 20, IL-1β + SenA 40 μg/mL group and IL-1β + SenA 80 μg/mL group. To confirm the effects of SenA on IL-1β-stimulated chondrocytes through the NLRP3 signalling pathway, the NLRP3 activator, nigericin (Nig, 15 μmol/L, MedChemExpress, Monmouth Junction, NJ) was used to treat chondrocytes. Chondrocytes were divided into four groups: control group, IL-1β group, IL-1β + SenA (80 μg/mL) group and IL-1β + SenA + Nig group. SenA was dissolved in 0.1% dimethyl sulphoxide (DMSO). In all cell cultures, the concentration of DMSO was less than 0.05%, which had no perceptible impact on cell growth or death.

### Cell viability analysis

The treated chondrocytes (5 × 10^3^/mL) were inoculated in 96-well plate. Subsequently, 20 μL MTT solution (5 mg/mL, Sigma-Aldrich, St. Louis, MO) was added to each well. After 4 h, each well was added with 200 μL DMSO. The 96-well plate was placed on a micro-oscillator to promote crystallization to dissolute for 20 min. The absorbance was measured at 490 nm by a microplate reader (Olympus, Tokyo, Japan).

### Cell proliferation analysis

The treated chondrocytes (2 × 10^5^ cells/well) were seeded into 24-well plates and incubated with 10 μM EdU solution (Thermo Fisher Scientific, Carlsbad, CA) for 2 h. Subsequently, the chondrocytes were immobilized with 4% formaldehyde solution for 15 min, washed three times with phosphate buffer saline (PBS), and penetrated with 0.4% Triton X-100 for 20 min at room temperature. Ultimately, the chondrocytes were dyed with DAPI solution (Thermo Fisher Scientific, Carlsbad, CA) and incubated for 15 min at room temperature. The EdU-positive cells (red) ratio was recorded.

### Cell apoptosis analysis

The harvested chondrocytes were washed with PBS and rapidly resuspended in 500 μL conjugate buffer. Then, 10 μL Annexin V‐FITC and 10 μL propidium iodide (PI, BD Pharmingen, San Diego, CA) were added to buffer. The BD FACS flow cytometer (BD Biosciences, San Jose, CA) was used to analyse cell apoptosis.

### Mouse OA model

After seven days of acclimatization, 24 male C57BL/6 mice were divided into experimental (*n* = 18) and sham (*n* = 6) groups. In the experimental group, the medial meniscus tibial ligament (MMTL) was cut off at right knee incision to induce OA according to a previous study (Glasson et al. 2007). Briefly, mice were anesthetized with isoflurane inhalation (5% for deeply anaesthetization and 2–3% for surgery), and the right knee area was scraped and repealed to prepare for operation. After the skin incision was made on the medial side of knee, the medial patellar tendon joint capsule was incised, the subpatellar fat pad was peeled off bluntly, and the MMTL of the medial meniscus was visualized. The MMTL was cut off and resulted in destabilization of the medial meniscus (DMM). In the sham group, the MMTL was visible but not cut off. Finally, the joint capsule and skin both were closed by suture. One week after surgery, experimental mice were divided into three groups (*n* = 6 per group): OA group (no treatment), OA + SenA 20 mg/kg group (intraperitoneally injected with 20 mg/kg/d SenA for one week) and OA + SenA 40 mg/kg group (intraperitoneally injected with 40 mg/kg/d SenA for one week). The mice in sham group were only injected with commensurate normal saline. The overall health status of mice was monitored according to the ethical guidelines of the research.

### Histologic evaluation

After 4 weeks of continuous feeding, mice were intraperitoneally anaesthetized with 1% sodium pentobarbital and immediately sacrificed. Then, the knee joints were fixed in 4% paraformaldehyde for 24 h, and treated with ethylenediaminetetraacetic acid (EDTA) to decalcification for seven days. The joints were embedded in paraffin, and 7 mm sections were taken for haematoxylin–eosin (H&E) staining (Kim et al. 2020). Specifically, the paraffin sections were deparaffinized, alcohol hydration was graded, haematoxylin solution was stained for 20 min, and eosin solution was counterstained (Sigma-Aldrich, St. Louis, MO) for 10 min. After gradient ethanol dehydration, transparent and sealed, the image-Pro image analysis software (Media Cybernetics, Rockville, MD) was used to observe the pathological changes of articular cartilage.

### Western blot analysis

The chondrocytes and knee articular cartilage tissue were flushed three times with PBS and lysed using RIPA lysis buffer (Beyotime Biotechnology, Shanghai, China) for 30 min. The samples were separated by 10% sodium dodecyl sulphate-polyacrylamide gel electrophoresis (SDS-PAGE) and transferred to polyvinylidene fluoride (PVDF) membranes (Millipore, Billerica, MA). The membranes were blocked with 5% skim-milk powder for 30 min at room temperature and then incubated with the corresponding primary antibodies of NLRP3, ASC, caspase-1, IL-6, IL-18, TNF-α, MMP13, ADAMTS4/5, IGF-1, aggrecan and Col2a1 (1:1000, Sigma-Aldrich, St. Louis, MO) at 4 °C overnight. After washing with PBS, the membranes were incubated with horseradish peroxidase-labelled secondary antibody (1:2000, Cell Signaling Technology, Danvers, MA) for 1 h at room temperature. Ultimately, the intensity of protein expression was measured by an enhanced chemiluminescence reagent (Thermo Fisher Scientific, Carlsbad, CA). GAPDH served as control.

### Statistical analysis

All data were analysed by SPSS 18.0 (Chicago, IL). All above experiments were conducted in triplicate and all data were expressed as mean ± standard deviation (SD). Comparisons between groups were performed using one-way ANOVA with *post hoc* Tukey's test. *p* < 0.05 was regarded as statistically significance.

## Results

### SenA enhanced the cell viability of chondrocytes stimulated by IL-1β

**Figure 1.**
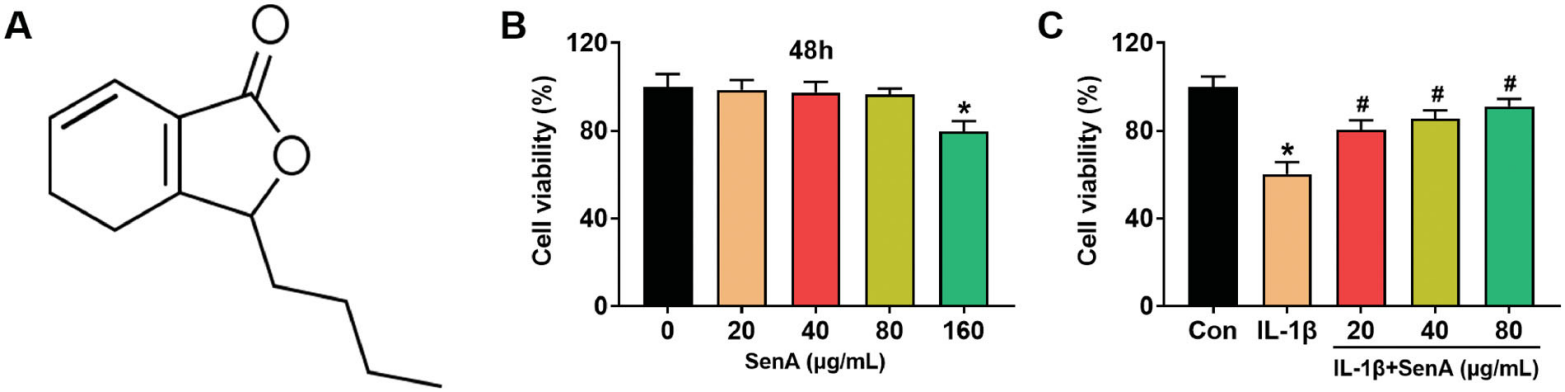
SenA enhanced cell viability of chondrocytes stimulated by IL-1β. (A) The chemical structure of SenA. (B) The cell viability of chondrocytes treated with different concentrations of SenA (0, 20, 40, 80 and 160 μg/mL) was measured by MTT assay. *p < 0.05. (C) The cell viability of IL-β (10 ng/mL) stimulated chondrocytes treated with SenA (20, 40 and 80 μg/mL) was measured by MTT assay. *p < 0.05 compared with control (con) group; ^#^*p* < 0.05 compared with IL-1β group.

The chemical structure of SenA is exhibited in Figure 1(A). To ascertain the cytotoxic effect of SenA on chondrocytes, we detected the cell viability of chondrocytes treated with gradient concentrations of SenA (0, 20, 40, 80 and 160 μg/mL) by MTT assay. The results indicated SenA had no notably cytotoxic effect on chondrocytes at concentrations of 0–80 μg/mL, while significantly attenuated the cell viability at the concentration of 160 μg/mL (Figure 1(B)). Therefore, the concentrations of SenA used in the following experiments were 20, 40 and 80 μg/mL. Furthermore, SenA significantly increases the viability of IL-1β-stimulated chondrocytes (Figure 1(C)). All those results indicated that SenA could enhance the cell viability of IL-1β-stimulated chondrocytes.

### The protective effect of SenA on IL-1β-stimulated chondrocytes

**Figure 2.**
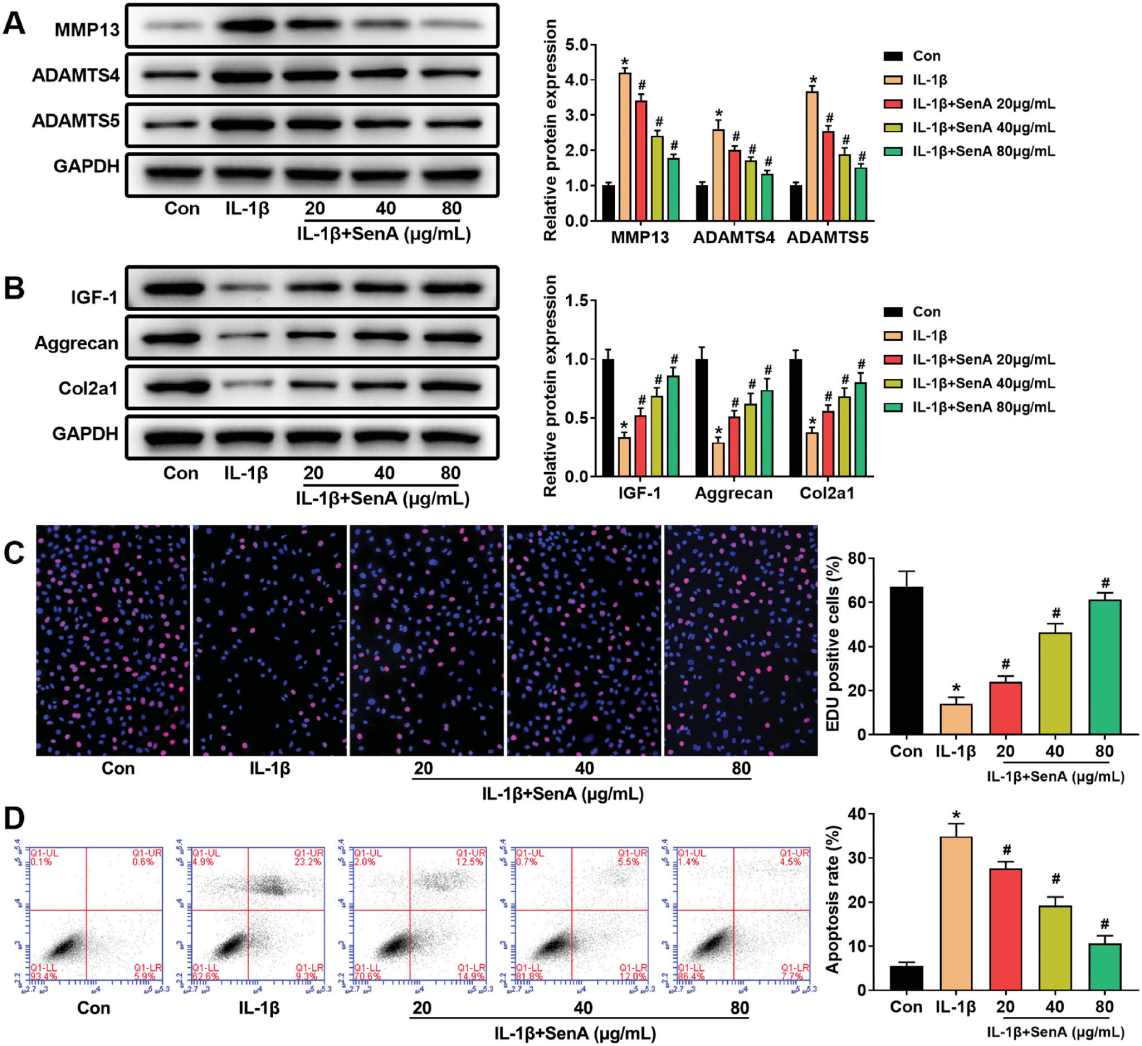
The protective effect of SenA on IL-1β-stimulated chondrocytes. (A) SenA inhibited the expression of catabolic marker proteins (MMP13, ADAMTS4/5) in IL-1β-stimulated chondrocytes. (B) SenA enhanced the expression of anabolic marker proteins (IGF-1, aggrecan and Col2a1) in IL-1β-stimulated chondrocytes. (C) SenA promoted cell proliferation of IL-1β-stimulated chondrocytes. (D) SenA inhibited cell apoptosis of IL-1β-stimulated chondrocytes. *p < 0.05 compared with control (con) group; ^#^*p* < 0.05 compared with IL-1β group.

To investigate the effect of SenA on the IL-1β-stimulated catabolic response in chondrocytes, the expression of catabolic marker proteins (MMP13, ADAMTS4/5) and anabolic marker proteins (IGF-1, aggrecan and Col2a1) were detected by western blot. IL-1β markedly increased the expression of MMP13, ADAMTS4 and ADAMTS5, the addition of SenA significantly inhibited MMP13 and ADAMTS4/5 expression (Figure 2(A)). Meanwhile, IL-1β markedly reduced the protein expression of IGF-1, aggrecan and Col2a1, which were blocked by SenA treatment (Figure 2(B)). These findings indicated that SenA could restrain the catabolic responses of chondrocytes stimulated by IL-1β.

Furthermore, we performed EdU assays and flow cytometric analysis to assess the effect of SenA on proliferation and apoptosis of chondrocytes. IL-1β markedly reduced cell proliferation and increased cell apoptosis compared to the control group, while the addition of SenA significantly inhibited IL-1β-stimulated apoptosis and promoted proliferation of chondrocytes (Figure 2(C,D)). Together, these data indicated that SenA exhibited anti-apoptotic and pro-proliferation effects on IL-1β-stimulated chondrocytes.

### SenA blocked the activation of NLRP3 signalling pathway

**Figure 3.**
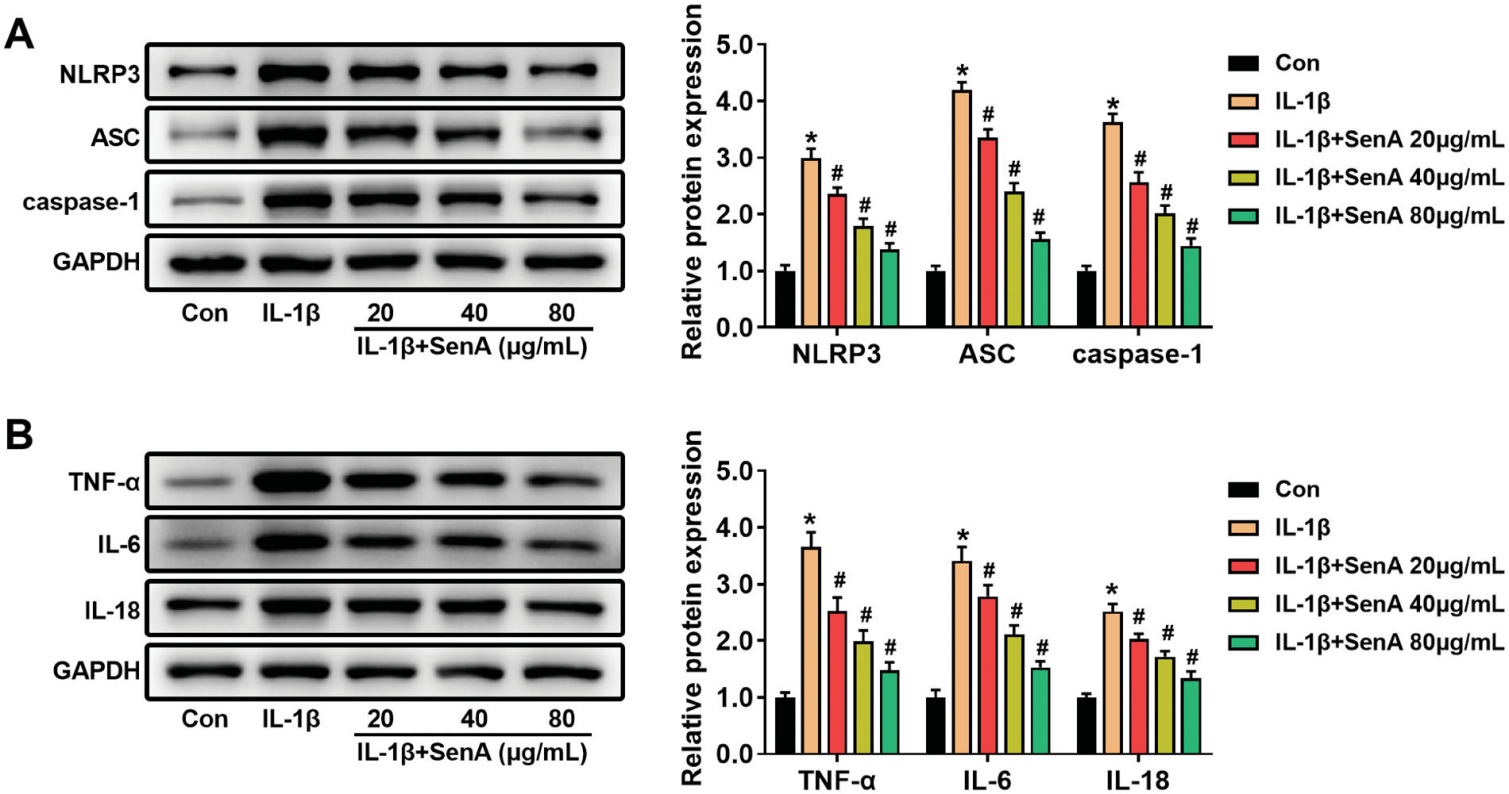
SenA blocked activation of the NLRP3 signalling pathway. (A) SenA inhibited the NLRP3 signalling pathway-related proteins (NLRP3, ASC and caspase-1) in IL-1β-stimulated chondrocytes. (B) SenA inhibited the protein expression of inflammatory cytokines (TNF-α, IL-6 and IL-18) in IL-1β-stimulated chondrocytes. *p < 0.05 compared with control (con) group; ^#^*p* < 0.05 compared with IL-1β group.

Previous studies reported that NLRP3 signalling pathway plays a key role in the mechanism of OA (Yang et al. 2020). To explore the effect of SenA on the NLRP3 signalling pathway in IL-1β-stimulated chondrocytes, the levels of the inflammatory mediators related to NLRP3 inflammasome were analysed by western blot. SenA decreased the levels of NLRP3, ASC and caspase-1 that was induced by IL-1β (Figure 3(A)). Furthermore, we detected the effect of SenA on the levels of inflammatory cytokines TNF-α, IL-6 and IL-18. The levels of TNF-α, IL-6 and IL-18 in IL-1β-stimulated chondrocytes were reduced by SenA (Figure 3(B)). These results indicated that SenA blocked the NLRP3 signalling pathway, thereby inhibiting IL-1β-stimulated inflammation in chondrocytes.

### NLRP3 agonist nigericin eliminated the protective effect of SenA on chondrocytes

**Figure 4.**
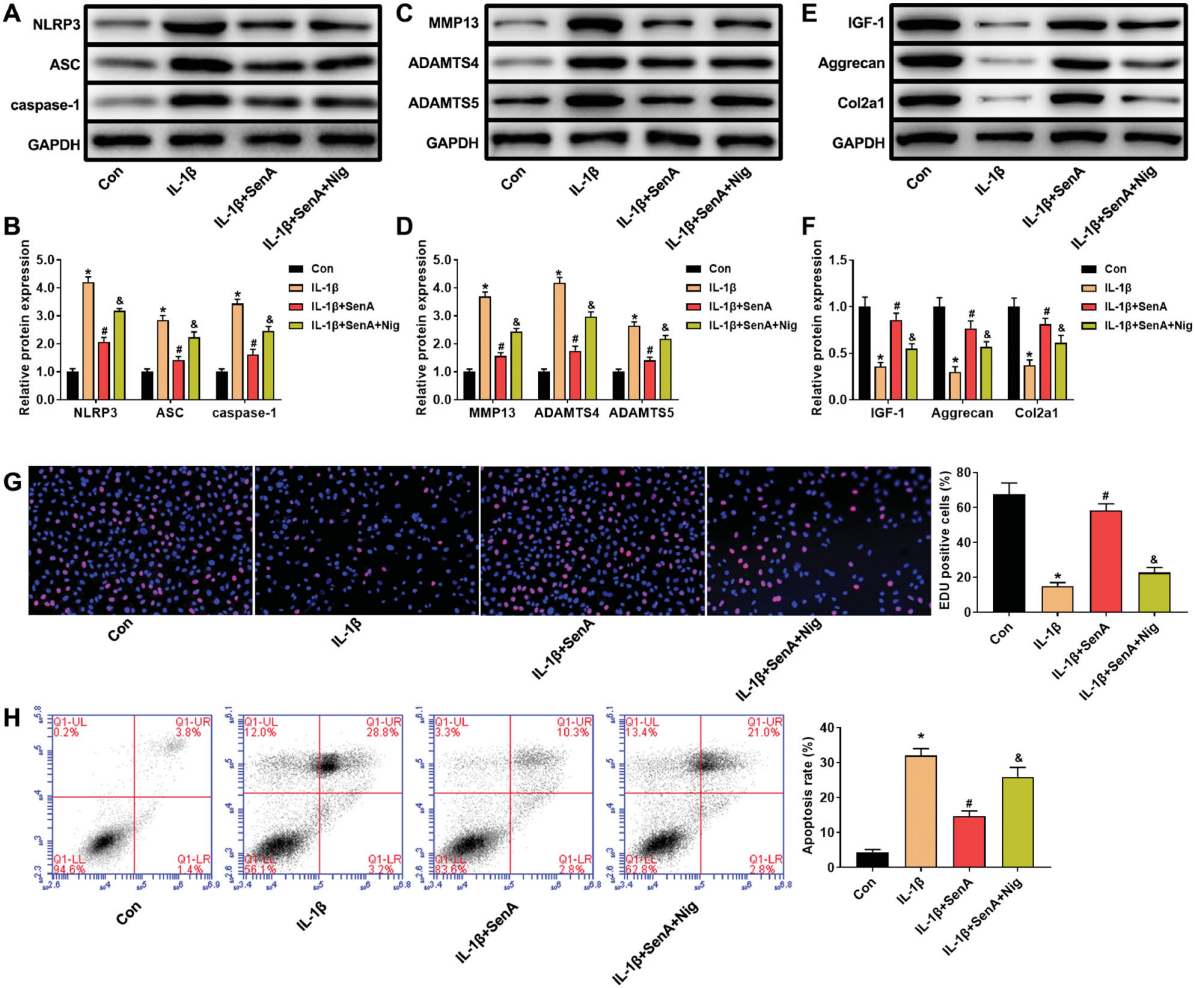
NLRP3 agonist nigericin eliminated the protective effect of SenA on chondrocytes. (A, B) Nigericin enhanced the protein expression of NLRP3 signalling pathway-related proteins (NLRP3, ASC and caspase-1) in IL-1β-stimulated chondrocytes. (C, D) Nigericin enhanced the expression of catabolic marker proteins (MMP13, ADAMTS4/5) in IL-1β-stimulated chondrocytes. (E, F) Nigericin inhibited the expression of anabolic marker proteins (IGF-1, aggrecan and Col2a1) in IL-1β-stimulated chondrocytes. (G) Nigericin inhibited cell proliferation of chondrocytes. (H) Nigericin promoted cell apoptosis of chondrocytes. *p < 0.05 compared with control (con) group; ^#^*p* < 0.05 compared with IL-1β group; ^&^*p* < 0.05 compared with IL-1β + SenA group.

To confirm the protective effect of SenA on the NLRP3 signalling pathway in IL-1β-stimulated chondrocytes, SenA (80 μg/mL) and NLRP3 agonist (nigericin) were used to treat the IL-1β-stimulated chondrocytes. The expression levels of NLRP3, ASC, caspase-1, MMP13, ADAMTS4 and ADAMTS5 in IL-1β-stimulated chondrocytes were decreased by SenA treatment (Figure 4(A–D)). In addition, SenA increased the levels of IGF-1, aggrecan and Col2a1 in IL-1β-stimulated chondrocytes (Figure 4(E,F)). However, nigericin significantly increased the levels of NLRP3, ASC, caspase-1, MMP13, ADAMTS4 and ADAMTS5, and decreased the levels of IGF-1, aggrecan and Col2a1 (Figure 4(A–F)).

In addition, we detected apoptosis and proliferation of chondrocytes after treatment with SenA and nigericin. The EdU-positive cells in IL-1β + SenA + Nig group were significantly less than that of IL-1β + SenA group, indicating that nigericin inhibited the proliferation of chondrocytes (Figure 4(G)). Nigericin treatment significantly promoted apoptosis of chondrocytes compared to IL-1β + SenA group (Figure 4(H)). Together, these results suggested that SenA plays a protective role in mice chondrocytes by inhibiting NLRP3 signalling pathways.

### SenA alleviated articular cartilage destruction by inhibiting NLRP3 signalling pathway in OA mice

**Figure 5.**
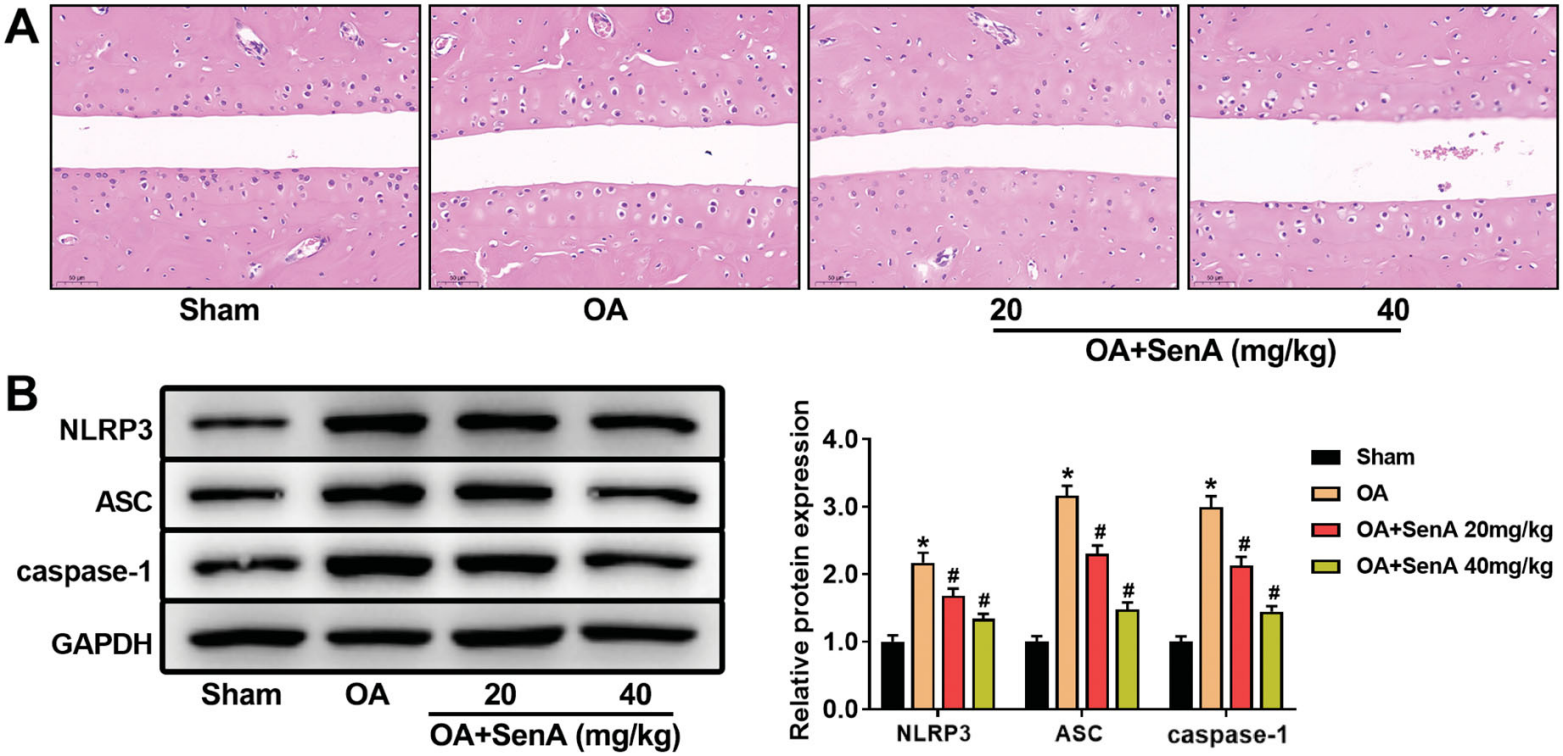
SenA alleviated articular cartilage destruction in OA mice by inhibiting the NLRP3 pathway. (A) H&E staining was used to observe the pathological changes of knee joint. (B) SenA inhibited NLRP3 signalling pathway-related proteins (NLRP3, ASC and caspase-1) in OA mice. *p < 0.05 compared sham group; ^#^*p* < 0.05 compared with OA group.

To further confirm the protective effect of SenA against OA progression *in vivo*, we established the mice OA model. H&E staining showed serious erosion of cartilage, thinner cartilage layers, evident roughness surfaces, and cartilage destruction in mice OA model. However, SenA-treated mice displayed less damage (Figure 5(A)). Furthermore, we detected the levels of NLRP3 signalling pathway related protein by Western blot. SenA significantly reduced the levels of NLRP3, ASC and caspase-1 in (Figure 5(B)), which is consistent with the results of *in vitro* experiments. Collectively, these results indicated that SenA could alleviate the progression of OA by inhibiting NLRP3 signalling pathway.

## Discussion

OA is the main reason of chronic disability in the elderly, with about 18% of women and 10% of men suffering from symptomatic OA (Mandl 2019). A population-based cohort study showed that the lifetime risk of patients with symptomatic knee OA was approximately 45% (Tang et al. 2016). There are currently no effective drugs to treat OA disease or slow its progression. Although existing drugs such as acetaminophen, duloxetine, steroidal and non-steroidal anti-inflammatory drugs can relieve pain of OA patients, they cannot cure OA due to serious adverse reactions and poor efficacy (Zhu et al. 2018). In search of more effective drugs for OA, plant natural products with anti-inflammatory and hypotoxicity have attracted increasing attention, including *chuanxiong* (Ye et al. 2011). Therefore, this study focussed on the role of SenA, one of the main components isolated from *Ligusticum chuanxiong*.

Inflammatory cytokines are responsible for the reduction of metabolic homeostasis in the tissues that form joints by accelerating catabolism and destruction processes (Jhun et al. 2021; Wang et al. 2021). Additionally, they affect the production of cytokines, enzymes and other inflammatory cytokines, such as TNF-α, IL-6, IL-1β and IL-18 through intracellular signal transduction pathways (Wojdasiewicz et al. 2014). In this study, we found that SenA significantly decreased the levels of TNF-α, IL-6 and IL-18 in IL-1β-stimulated chondrocytes, indicating SenA prevented the progression of OA by inhibiting levels of inflammatory cytokines.

A large amount of evidence showed that IL-1β induces strong inflammatory response and promotes the degradation of ECM (Wang et al. 2019). IGF-1, Col2a1 and aggrecan, secreted by chondrocytes, are important components of ECM and play vital role in maintaining the physiological function of articular cartilage (Ni et al. 2015). Matrix metalloproteinases (MMPs, such as MMP13) and a disintegrin and metalloproteinase with thrombospondin motifs (ADAMTSs, such as ADAMTS4/5) are the main enzymes that promote cartilage destruction and ECM degradation (Zhang et al. 2019). Additionally, ADAMTS4/5 can degrade aggrecan in the absence of MMPs (Malfait et al. 2002). Consistent with previous studies (Fei et al. 2019), our data showed that IL-1β (10 ng/mL) dramatically increased inflammatory levels, apoptosis and the levels of catabolic processes-related proteins (MMP13, ADAMTS4/5), whereas inhibited proliferation and the levels of anabolisms-related proteins (IGF-1, Col2a1 and aggrecan) of mice chondrocytes. However, SenA inhibited the effect of IL-1β on chondrocytes. These results indicated that SenA exhibited the protective effect in IL-1β stimulated chondrocytes, which may provide a new mechanism in the treatment of OA.

NLRP3 inflammasome has been reported to be involved in the pathogenesis and progression of OA (McAllister et al. 2018). Some studies have reported the connection between the mechanisms of plant natural products alleviating OA and the NLRP3 signalling pathway. For example, Zu et al. (2019) reported that icariin suppressed OA via inhibiting inflammatory necrosis mediated by NLRP3/caspase-1 signalling in OA models *in vitro* and *in vivo*. Dong et al. reported that sinomenine inhibits cell apoptosis and decreased NLRP3 protein expression and inflammatory levels of IL-1β-stimulated chondrocytes (Dong et al. 2019). In this study, we found that SenA inhibited the NLRP3 signalling pathway in IL-1β-stimulated chondrocytes and OA mice. SenA treatment markedly reduced the expression levels of NLRP3 signalling pathway-related proteins (NLRP3, ASC and caspase-1). Furthermore, NLRP3 agonist nigericin eliminated the protective effect of SenA on chondrocytes. These data suggested that SenA alleviated OA progression by inhibiting the NLRP3 signalling pathway.

SenA alleviated the progression of OA by inhibiting NLRP3 signalling pathways, which may bring new prospects for OA prevention or treatment.

## Authors contributions

Minglei Shao designed the study and wrote the paper; Dongwei Lv and Kai Zhou provided technical support and obtained data; Haijun Sun and Zhitao Wang analysed data.

## Data Availability

All data of this manuscript used to support the findings of this study may be released upon application to the correspondence author.
