# Correction

**DOI:** 10.1080/13880209.2022.2113958

**Published:** 2022-08-29

**Article title**: Senkyunolide A inhibits the progression of osteoarthritis by inhibiting NLRP3 signaling pathway

**Authors**: Shao, M., Lv, D., Zhou, K., Sun, H., & Wang, Z.

**Journal**: *Pharmaceutical Biology*

**Bibliometrics**: Volume 60, Number 01, pages 535–542

**DOI**: https://doi.org/10.1080/13880209.2022.2042327

The authors have found an error in the body weight of Male C57BL/6 mice represented in the sub-section “Animals” under the “Materials and methods” section of the article, where it has been mistakenly written as “200–220 g” instead of “20–22 g”.

The sentence “Male C57BL/6 mice (200–220 g) were purchased from the Animal Center of the Chinese Academy of Sciences” is now corrected as Male C57BL/6 mice **(20–22 g)** were purchased from the Animal Center of the Chinese Academy of Sciences.

